# Cardiac atrophy associated to cancer: new perspectives in signaling pathways

**DOI:** 10.1186/s43556-025-00374-4

**Published:** 2025-11-25

**Authors:** Andrea C. Lodeiro, Saúl Leal-López, Silvia Costas-Abalde, Lucía Debasa-Corral, María J. Otero-Fraga, José Vilar, Hafid Ait-Oufella, Yolanda Pazos, Jesus P. Camiña, Icía Santos-Zas

**Affiliations:** 1 Grupo de Miología, IDIS, CHUS, SERGAS, Santiago de Compostela, Spain; 2 Grupo de Investigación Traslacional en Enfermedades del Aparato Digestivo (GITEAD), IDIS, CHUS, SERGAS, Santiago de Compostela, Spain; 3 Paris Cardiovascular Research Center Université Paris Cité, INSERM U970, Paris, France

Dear Editor,

Cancer cachexia is a devastating syndrome characterized by an ongoing weakening and loss of skeletal muscle, with an important impact on tolerance to anti-tumour therapies and responsible for 20–30% of cancer-related deaths. Such disorder is not restricted to skeletal muscle, but is a multifactorial pathology that also affects the heart, triggering the loss of cardiac muscle mass and leading to myocardial dysfunction [1]. The molecular mechanisms that cause cancer-associated cardiac atrophy are currently not known. Early studies postulated the decrease in protein synthesis and the increase in protein degradation as main executors of myocardial wasting. However, it has not been possible to clarify the main signaling pathway that controls protein degradation or whether the suppression of protein synthesis plays a relevant role in cardiac muscle loss [1]. The present study describes the underlying mechanisms of cardiac muscle atrophy in cancer using three preclinical tumour models: (i) orthotopic-model of pancreatic ductal adenocarcinoma (ORTHO-PDAC); (ii) genetically engineered mouse model of PDAC (GE-PDAC); and, (iii) Lewis lung carcinoma (LLC) model.

Phenotypically, body weight and cardiac mass decrease in all tumour-bearing mice (Fig. 1a). Tumour effect on anabolic and catabolic signaling pathways was assessed by immunoblot. Downregulation of mammalian target of rapamycin (mTOR) activity [pmTOR(S2448)], master regulator of protein synthesis, was observed in hearts of the ORTHO-PDAC model (Fig. 1b). This was supported by decreased phosphorylation of the mTOR downstream targets 4E-binding protein 1 [p4EBP1(T37/46)] and ribosomal protein S6 [pS6(S235/236)]. In contrast, upregulation of such proteins was observed in the GE-PDAC and LLC models. Besides, glycogen synthase kinase 3 phosphorylation [pGSK3a(S21/9)] was only significantly increased in the LLC model, reflecting GSK3 regulation only in cardiac muscle of LLC-bearing mice (Fig. 1b).

**Fig. 1:**
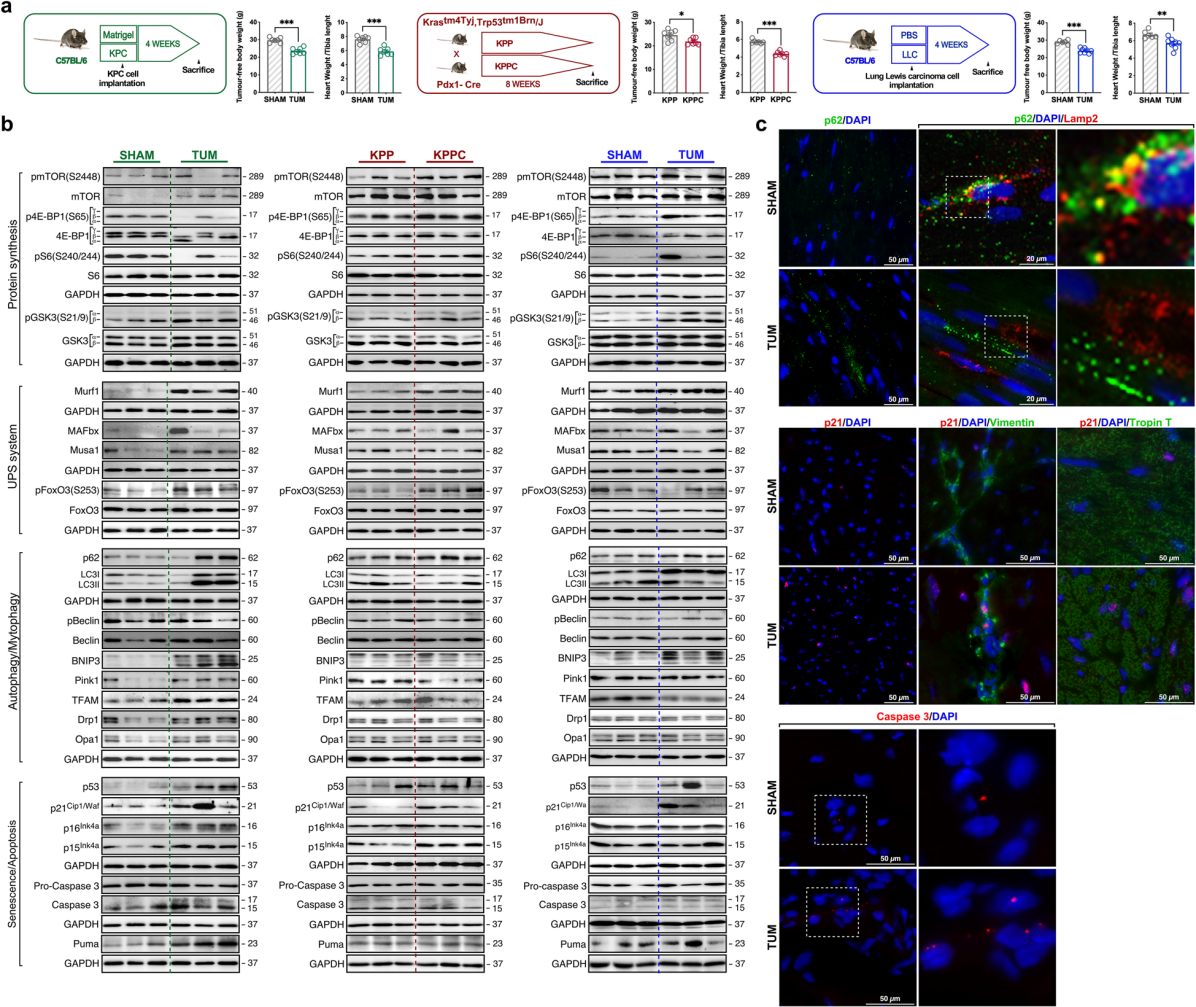
Tumour-dependent effect on cardiac muscle. **a** Overview of murine cancer cachexia models: orthotopic PDAC [sham and tumour transplanted mice (KPC cells; *n* = 6/group)], GE-PDAC [control KPP and tumour KPPC (*n* = 7/group)] and LLC [sham and tumour transplanted mice (LLC cells; *n* = 6 and *n* = 7 respectively)]; Tumour-free body weight and heart weight normalized to tibia length. **b** Representative immunoblots of cardiac muscle expression of: pmTOR(S2448), mTOR, p4E-BP1(S65), 4E-BP1, pS6(S240/244), S6 pGSK3α(S21/9)/pGSK3β(S21/9), GSK3a/GSK3β (protein synthesis); Murf1, MAFbx, Musa1, pFoxO3(S253) and Foxo3 (UPS system); p62, LC3, pBeclin1(S15), Beclin, BNIP3, Pink1, TFAM, DRP1 and OPA1 (autophagy/mytophagy) p53, p21^Cip/Waf^, p16^Ink4a^, p15^Ink4a^, pro-Caspase 3, Caspase 3 and Puma (senescence/apoptosis). Immunoblots are representative of the mean value of each group. **c** Representative immunostaining for p62, Lamp2, p21, vimentin, Troponin T and caspase 3 in transverse cryosections of hearts of sham or ORTHO-PDAC-bearing mice (*n* = 4/group). Data are expressed as mean ± SEM. *, **, *** *p* < 0.05; *p* < 0.01; *p* < 0.001, respectively

Metabolic remodeling in energy-depleted states, such as cachexia, induces activation of the ubiquitin–proteasome system (UPS) and autophagy to promote protein catabolism [2]. Hyperactivation of both pathways are implicated in skeletal muscle wasting, although its role in cardiac atrophy is still debated [1]. Protein expression of the E3 ubiquitin-ligases Murf1, MAFbx and MUSA1 was used to determine the implication of UPS in cardiac muscle loss. Hearts from ORTHO- PDAC, GE- PDAC and LLC models displayed elevated expression of Murf1. Significant elevation of MAFbx and MUSA1 was observed only in ORTHO-PDAC model (Fig. 1b). Interestingly, the phosphorylation level of the transcription factor Forkhead box O3 [pFoxO3(S253)], regulator of atrogenes involved in protein degradation, was upregulated in orthotopic and GE-PDAC mice, ruling out its role in the atrophy program (Fig. 1b). Autophagy system was evaluated as an essential process contributing to muscle mass reduction. All three cachectic models showed signs of autophagy flux impairment, with pronounced augment of autophagy-related protein p62/SQSTM1 (Fig. 1b, c). A lower accumulation of microtubule-associated protein 1 light chain 3 (LC3II), essential protein for the biogenesis and maturation of autophagosomes, was displayed in the GE-PDAC and LLC models, in contrast to the ORTHO-PDAC model. No activation of Beclin1 [pBeclin1(S15)], positive regulator of autophagy, supports autophagic defects in cardiac muscle in all three models (Fig. 1b). Furthermore, p62-positive vesicles do not co-localize with LAMP2, protein required for fusion of lysosomes with autophagosomes, suggesting an impairment in lysosomal function (Fig. 1c). Our results associate cardiac atrophy with an increase in UPS activity in parallel to autophagy blockade. Proteasome-mediated protein hypercatabolism may potentially occur as a consequence of autophagy blockade in which protein and organelle engulfment exacerbate cardiac dysfunction [3]. Tumour effect on mitochondrial homeostasis, mechanism with an enormous impact in muscle wasting, was also assessed [4]. Protein levels of the mitophagy inducers Bcl-2 interacting protein 3 (BNIP3), PTEN-induced kinase 1 (Pink1) and the mitochondrial transcription factor A (TFAM), regulator of mitochondrial biogenesis, were increased in ORTHO-PDAC-bearing mice with no significant effect on the fission protein dynamin-related protein 1 (Drp1) and in the fusion protein optic atrophy type 1 (Opa1) (Fig. 1b). In contrast, no differences in the level of BNIP3 were observed in GE-PDAC and LLC models. The expression of Pink1 was significantly reduced in hearts from GE-PDAC mice with no variation in LLC mice. Decreases in cardiac muscle TFAM levels were observed in LLC model, with no change for GE-PDAC. Finally, reduced expression level of Opa1 was just showed in LLC mice (Fig. 1b). Thus, dysfunction in mitochondrial turnover was a common link between models but changes in mitochondrial protein content were variable.

To determine new signaling pathways related to cancer-associated cardiac wasting, the evaluation of senescence and apoptosis in the myocardium was carried out. Both mechanisms preserve tissue homeostasis and their dysregulation is observed in several pathologies. Notably, cardiac muscle samples from all tumour-bearing mice showed elevated levels of the cyclin-dependent kinases (CDK) p53, p21^Cip1/Waf^, p16^INK4^ and p15^INK4^, although with different expression patterns among models (Fig. 1b, c). Despite this, p21^Cip1/Waf^ is established as a common regulator of cardiac senescence regardless of tumour type, targeting the main cell populations of the myocardium: cardiomyocytes (Troponin T-positive cells), fibroblasts and endothelial cells (vimentin-positive cells) (Fig. 1c). Accumulation of senescent cells in the heart has been documented in cardiovascular diseases [5]. However, the presence of such cells in atrophic cardiac muscle associated to cancer has not currently been described. Senescent cells secrete inflammatory molecules, trait known as the senescence-associated secretory phenotype (SASP). Curiously, IL6 and TNFα, cytokines described as drivers of cachexia are key factors of the SASP. In this context, senescence cells could be postulated as the source of two of the main cytokines involved in muscle wasting. In addition, the relationship between senescence and protein degradation pathways could be established as a feedback loop: SASP could block autophagy, leading to increased degradation by UPS. The accumulation of damaged structures as a result of autophagy blockage would contribute to an increase in senescent cells. The use of senolitics, like dasatinib or quercetin, would prove the effect of senescence activation. Finally, the regulator of mitochondrial membrane permeabilization, PUMA, as well as the executioner caspase acting downstream of mitochondria, Caspase 3, were raised in the ORTHO-PDAC model, with no level modifications in GE-PDAC and LLC mice (Fig. 1b, c). These results suggest a possible dual effect of p21^Cip1/Waf^ protein as a protector and an inducer of apoptotic mechanisms. The type and characteristics of the tumour may determine that senescent cells are redirected to develop apoptosis in the cachectic heart.

The present work provides compelling evidence of the considerable heterogeneity in the molecular mechanisms associated to cardiac wasting regarding tumour type and biologic variables. Despite such complexities, different pathways controlling protein turnover and degradation have been identified as common signature in cardiac wasting in all models: UPS activation, impaired autophagic degradation and dysregulation in mitochondrial homeostasis. Particularly noteworthy is the senescence program activation in a tumour therapy-independent manner. Senescence emerges as a crucial mechanism in the pathogenesis of cancer associated cardiac atrophy for the first time, postulating itself as a new therapeutic target. The development of anabolic and anti-catabolic approaches has been one of the main objectives in the battle against muscle wasting associated to cancer, focusing just on the molecular mechanisms affected in skeletal muscle. In view of the present results, cardiac muscle wasting mechanisms do not completely mimic molecular processes in cachectic skeletal muscle, and may even be opposite as in the case of protein synthesis. This is a crucial consideration for the development of therapeutic strategies. This study underlines the need to move towards much more personalised therapies that take into account the characteristics and type of tumour and the proper behaviour of cardiac muscle.

## Supplementary Information

Supplementary Material 1.

Supplementary Material 2.

## Data Availability

The data that support the findings of this study are available from the corresponding author upon reasonable request.
