# Supplementary material for: Cardiac atrophy associated to cancer: new perspectives in signaling pathways
Source: Mol Biomed. 2025 Nov 25;6:118. doi: 10.1186/s43556-025-00374-4 (PMC12647403; doi:10.1186/s43556-025-00374-4)
Supplement: Supplementary file 1 — Supplementary Material 1. [file 43556_2025_374_MOESM1_ESM.docx]

**Cardiac atrophy associated to cancer: new perspectives in signaling pathways**

Andrea C. Lodeiro^1,2#,^ Saúl Leal-López^2#^, Silvia Costas-Abalde^1,2^, Lucía Debasa-Corral^1^, María J. Otero-Fraga^2^, José Vilar^3^, Hafid Ait-Oufella^3^, Yolanda Pazos^2^, Jesus P. Camiña^1^ and Icía Santos-Zas^1^

(1) Grupo de Miología, IDIS, CHUS, SERGAS, Santiago de Compostela, Spain.

(2) Grupo de Investigación Traslacional en Enfermedades del Aparato Digestivo (GITEAD), IDIS, CHUS, SERGAS, Santiago de Compostela, Spain.

(3) Paris Cardiovascular Research Center Université Paris Cité, INSERM U970, Paris, France.

# This authors contributed equally.

Corresponding author: Icia Santos Zas. [icia.santos@rai.usc.es](mailto:icia.santos@rai.usc.es)

**Materials and methods**

**Preclinical cancer cachexia models.** Animals were maintained under specific pathogen-free conditions and handled in accordance with the Animal Welfare guidelines. All protocols and experiments were aproved by the Bioethics Committee at the University of Santiago de Compostela (license number 15012/2023/013).

*(i) Orthotopic pancreatic ductal adenocarcinoma (PDAC) model:* C57BL6/J male mice (8 weeks) were injected with 3x10E3 murine pancreatic cancer cells KPC FC1245, from here on referred as KPC, suspended in Matrigel (tumour) or equal volume of Matrigel (sham) into the tail of the pancreas; KPC cells were generated in the Tuveson-laboratory (Cold Spring Harbor Laboratory, New York, USA). These cells were isolated from PDA tumor of Kras^G12D/+^; p53^R17H/+^; Pdx1-Cre mice of C57BL/6 background. *(ii) Genetically engineered PDAC (GE-PDAC) model:* Kras^tm4Tyj^, Trp53^tm1Brn^/J, and Pdx1-Cre mice strains, (The Jackson Laboratory, Bar Harbor, ME, US), were interbred to generate KPPC (tumour) and KPP (littermates lacking Pdx1-Cre; control) mice; and, *(iii) Lung Lewis Carcinoma (LLC) model:* C57BL/6 male mice (8 weeks) were inoculated with 1x10E6 LLC cells (ATCC, CRL-1642) suspended in phosphate buffered saline, PBS (tumour) or equal volume of PBS (sham) subcutaneously at the right flank. Orthotopic PDAC and LLC mice were euthanized 28 days after tumour cell inoculation. GE-PDAC mice were euthanized at 8 weeks due to rapid tumour progression and mortality. The murine models used in this work were chosen as candidates for the study of cardiac wasting because their reproducibility, speed and because they are well-stablished cachectic models. Specifically, pancreatic models are highly associated with the development of cachexia.

**Immunoblot analysis**. Heart samples were directly lysed in in ice-cold RIPA buffer [50 mM Tris-HCl (pH 7.2), 150 mM NaCl, 1 mM EDTA, 1% (v/v) NP-40, 0.25% (w/v) Na-deoxycholate, protease inhibitor cocktail (Sigma, St. Louis, MO, US), phosphatase inhibitor cocktail (Sigma)]. The lysates were clarified by centrifugation (14,000xg for 15 min at 4°C) and the protein concentration was quantified using the QuantiProTM BCA assay kit (Sigma). For immunoblotting, equal amounts of protein were fractionated by SDS-PAGE and transferred onto nitrocellulose membranes. Immunoreactive bands were detected by enhanced chemiluminescence (Thermo Fisher Scientific, Pierce, Rockford, IL, US).

**Immunofluorescence analysis.** Hearts were excised, rinsed in PBS, and frozen in liquid nitrogen. They were cut along their length into 7-μm thick cardiac muscle cryosections. Heart sections for immunofluorescence analysis were fixed with paraformaldehyde 4%, permeabilized using 0.2% Triton X100 in Phosphate Buffer Solution (PBS), blocked with PBS-T (0.2% Triton X100, 10% goat serum, 0.2% BSA in PBS), and incubated with primary antibodies diluted in PBS-T. Finally, sections were incubated with a mixture of appropriate secondary antibodies.

**Statistics.** The data were analyzed with the GraphPad Prism processing program. The results are displayed as mean ± SEM. Differences between values were examined using the non-parametric Mann-Whitney test and they were considered significant at p < 0.05 (p* < 0.05; p** < 0.01; p*** < 0.001)

**Table 1. Primary antibodies**

| **Primary Antibody** | **Use** | **Dilution** | **Supplier** | **Reference** |
| --- | --- | --- | --- | --- |
| **pmTOR** | WB | 1:500 | Cell Signaling | 5536 |
| **mTOR** | WB | 1:500 | Cell Signaling | 2972 |
| **p4EBP1 (T37/46)** | WB | 1:2000 | Cell Signaling | 9451 |
| **4EBP1** | WB | 1:2000 | Cell Signaling | 9452 |
| **pS6(235/236)** | WB | 1:2000 | Cell Signaling | 2211 |
| **S6** | WB | 1:2000 | Cell Signaling | sc-74459 |
| **pGSK3𝛂/β** | WB | 1:2000 | Cell Signaling | 9331 |
| **GSK3𝛂/β** | WB | 1:2000 | Santa Cruz Bio. | sc-56913 |
| **Murf1** | WB | 1:500 | Santa Cruz Bio. | sc-398608 |
| **MAFbx** | WB | 1:500 | Santa Cruz Bio. | sc-166806 |
| **MUSA1** | WB | 1:500 | Santa Cruz Bio. | sc-514862 |
| **pFoxO3(S253)** | WB | 1:1000 | Cell Signaling | 13129 |
| **FoxO3** | WB | 1:1000 | Cell Signaling | 2497 |
| **p62/SQSTM1** | WB | 1:1000 | Cell Signaling | 5114 |
| **p62/SQSTM1** | IF | 1:400 | Cell Signaling | 23214 |
| **LC3A/B** | WB | 1:2000 | Cell Signaling | 12741 |
| **pBeclin1(S15)** | WB | 1:500 | Cell Signaling | 84966 |
| **Beclin1** | WB | 1:500 | Cell Signaling | 3495 |
| **BNIP-3** | WB | 1:2000 | Santa Cruz Bio. | sc-56167 |
| **Pink1** | WB | 1:500 | Santa Cruz Bio. | sc-517353 |
| **TFAM** | WB | 1:1000 | Santa Cruz Bio. | sc-16965 |
| **Drp1** | WB | 1:1000 | Cell Signaling | 8570 |
| **Opa1** | WB | 1:1000 | Cell Signaling | 80471 |
| **p21^Cip/Waf^** | WB | 1:500 | Cell Signaling | 64016 |
| **p21^Cip/Waf^** | IF | 1:500 | Abcam | ab188224 |
| **p53** | WB | 1:500 | Santa Cruz Bio. | sc-126 |
| **p16^Ink4a^** | WB | 1:1000 | Cell Signaling | 4824 |
| **p15^Ink4a^** | WB | 1:2000 | Antibodies.com | A94969 |
| **PUMA** | WB | 1:1000 | Cell Signaling | 98672 |
| **Caspase 3** | WB | 1:500 | Cell Signaling | 9662 |
| **Caspase 3** | IF | 1:250 | Cell Signaling | 9662 |
| **Vimentine** | IF | 1:500 | Abcam | ab8978 |
| **Troponin T** | IF | 1:500 | Abcam | ab8295 |

**Aditional References**

Huo Z, Chong F, Li N, Luo S, Yin L, Liu J,et al; Investigation on Nutrition Status and Clinical Outcome of Common Cancers (INSCOC) Group. Diagnostic Criteria for Cancer-Associated Cachexia: Insights from a Multicentre Cohort Study. J Cachexia Sarcopenia Muscle. 2025 doi: 10.1002/jcsm.13703.

**Abbreviations**

**4EBP1:** eukaryotic translation initiation factor 4E-binding protein 1; **CDK:** cyclin-dependent kinase; **Drp1:** dynamin-1-like protein; **Fox0:** forkhead box protein; **GE-PDAC:** genetically engineered pancreatic ductal adenocarcinoma; **GSK3:** glycogen synthase kinase 3; **IF:** immunofluorescence; **LC3:** microtubule-associated proteins 1A/1B light chain 3B; **LLC:** Lewis lung carcinoma; **MAFbx**: muscle Atrophy F-box/Atrognin-1; **MFI:** mean intensity value; **mTOR:** mammalian target of rapamycin; **Murf1:** muscle Ring-Finger Protein-1; **Musa1:** **F-box only protein 30; Opa1:** optic atrophy 1 protein; **PDAC:** pancreatic ductal adenocarcinoma; **Pink1:** PTEN-induced kinase 1; **PUMA:** p53 upregulated modulator of apoptosis; **S6:** ribosomal protein S6; **TFAM:**mitochondrial transcription factor A; **UPS:** ubiquitination-Proteasome System; **WB:** Western Blot
