# Supplementary material for: Cardiac atrophy associated to cancer: new perspectives in signaling pathways
Source: Mol Biomed. 2025 Nov 25;6:118. doi: 10.1186/s43556-025-00374-4 (PMC12647403; doi:10.1186/s43556-025-00374-4)
Supplement: Supplementary file 2 — Supplementary Material 2. [file 43556_2025_374_MOESM2_ESM.pdf]

Cardiac atrophy associated to cancer: new perspectives in signaling pathways

<u>Western Blot raw images and analysis</u>

Orthotopic-model of Pancreatic Ductal Adenocarcinoma (ORTHO-PDAC MODEL)

pmTOR

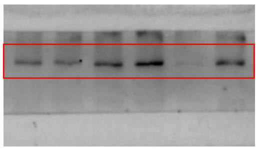

mTOR

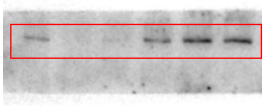

p4EBP1

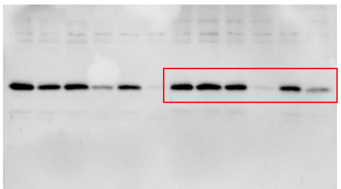

4EBP1

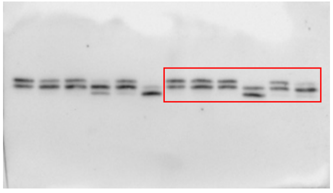

pS6

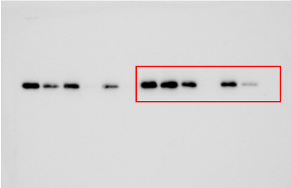

S6

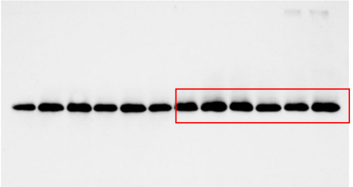

GAPDH

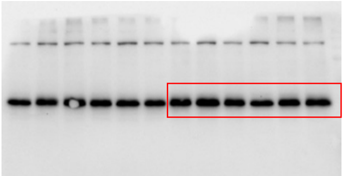

pGSK3

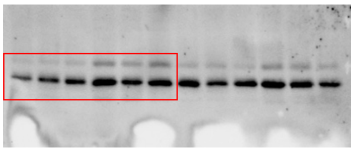

GSK3

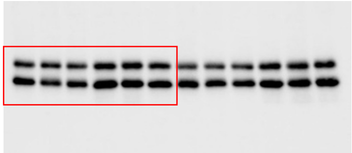

GAPDH

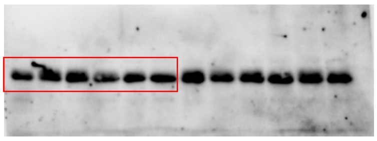

MURF1

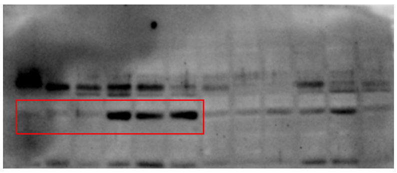

GAPDH

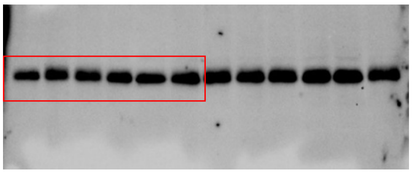

Mafbx

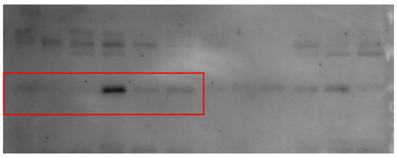

Musa

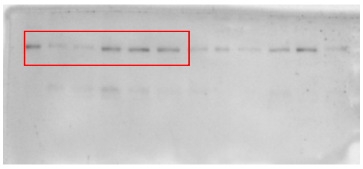

GAPDH

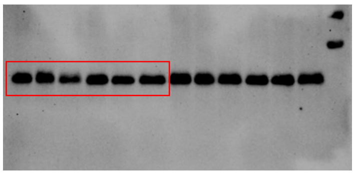

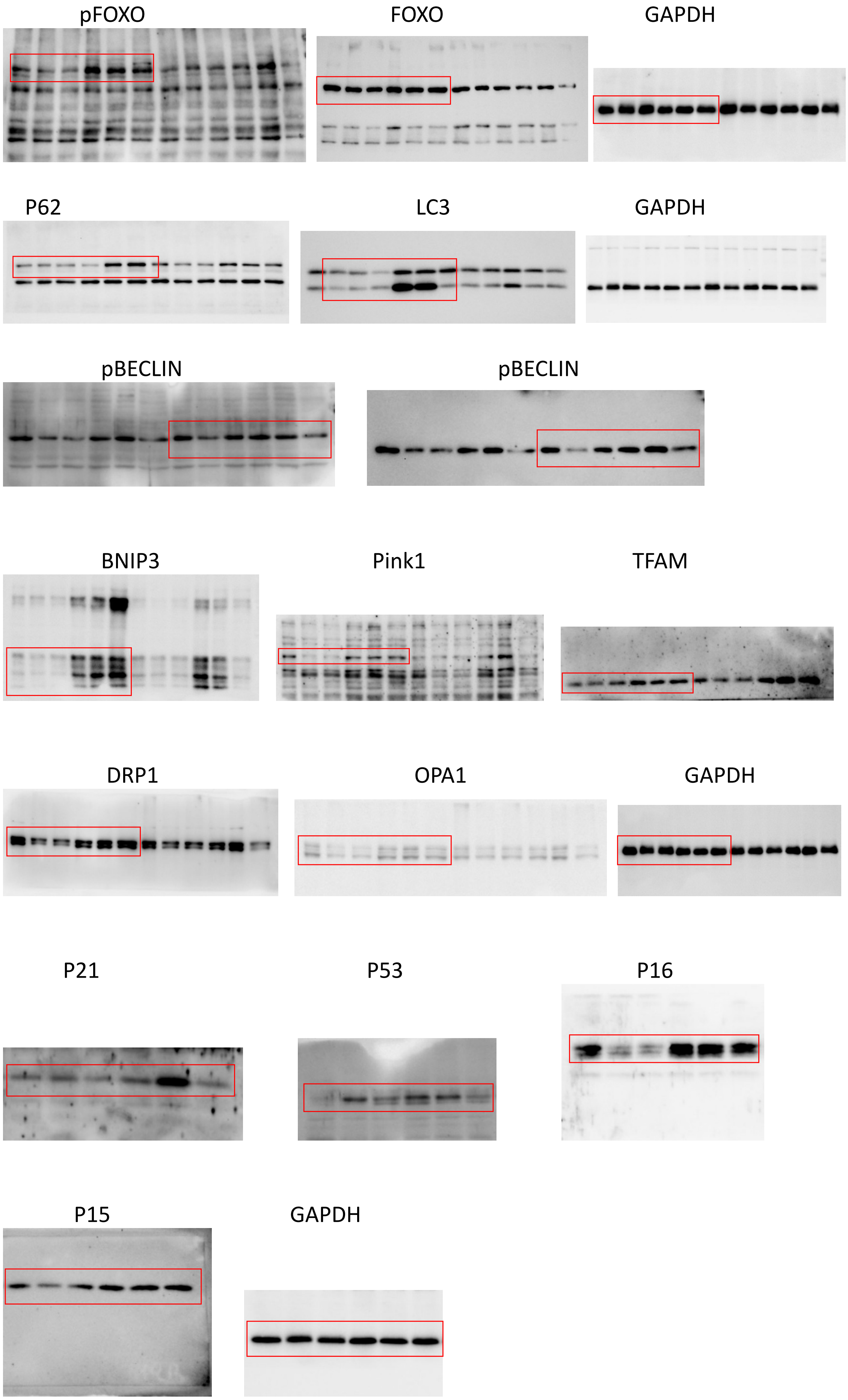
pFOXO
FOXO
GAPDH
P62
LC3
GAPDH
pBECLIN
pBECLIN
BNIP3
Pink1
TFAM
DRP1
OPA1
GAPDH
P21
P53
P16
P15
GAPDH

Procaspase 3

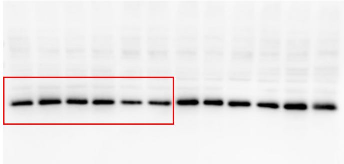

Caspase3

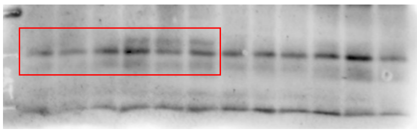

GAPDH

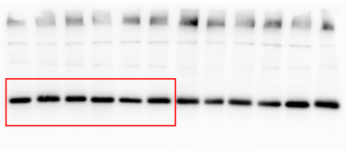

Puma

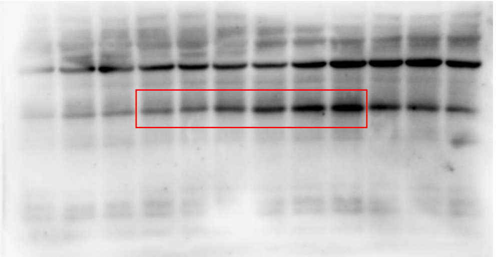

GAPDH

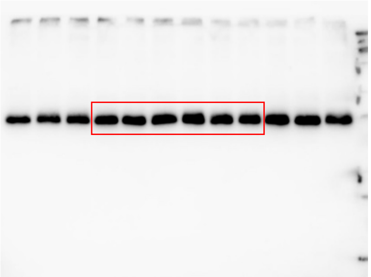

Genetically engineered mouse model of Pancreatic Ductal Adenocarcinoma (GE-PDAC MODEL)

pMTOR

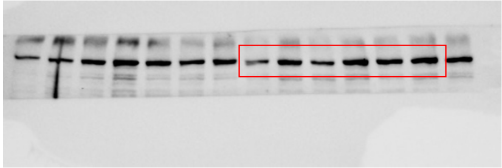

pmTOR

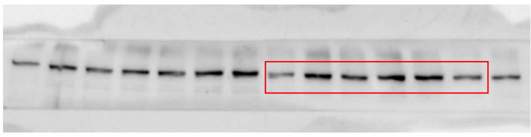

p4EBP1

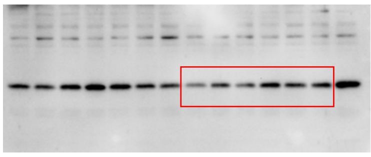

4EBP1

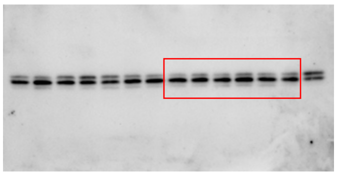

pS6

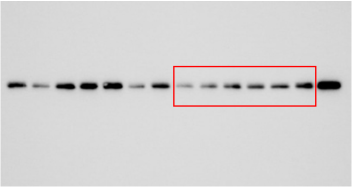

S6

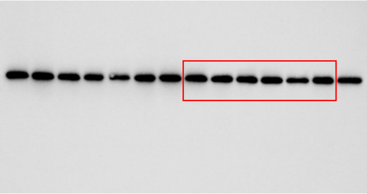

GAPDH

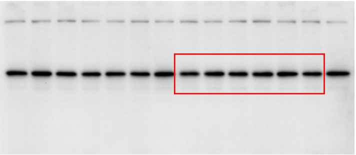

pGSK3

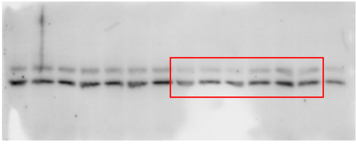

GSK3

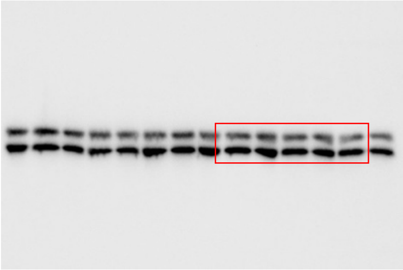

GAPDH

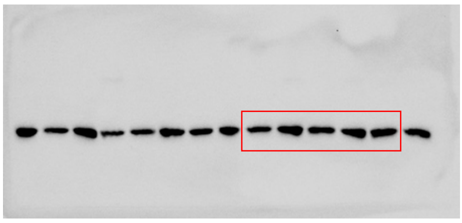

Murf1

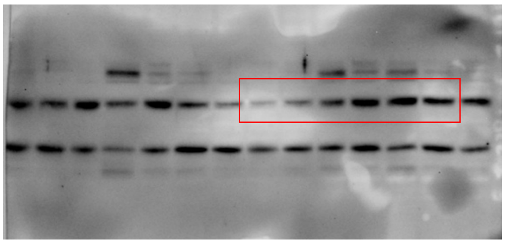

GAPDH

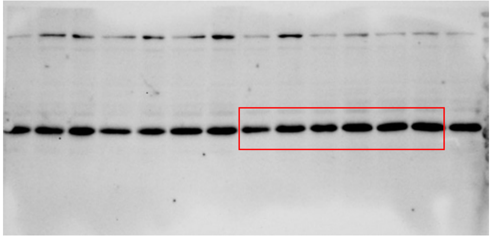

Mafbx

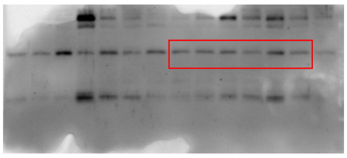

Musa 1

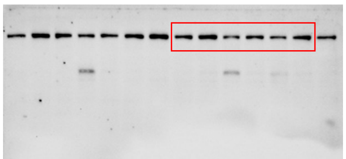

GAPDH

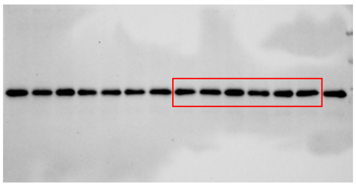

pFOXO

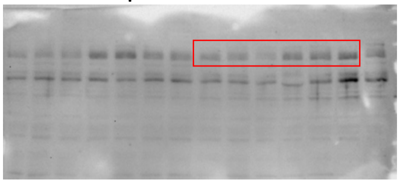

FoxO

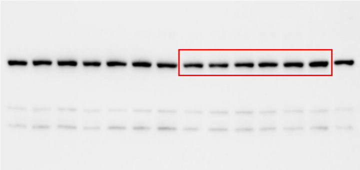

GAPDH

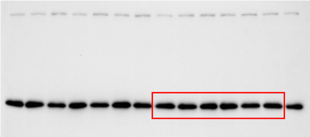

P62

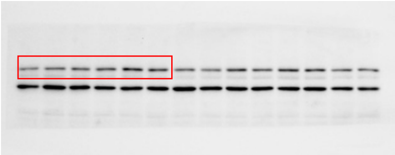

LC3.

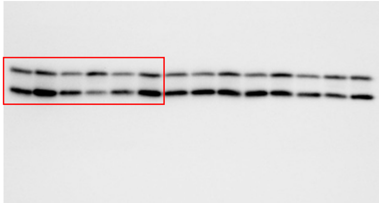

GAPDH

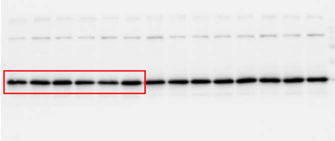

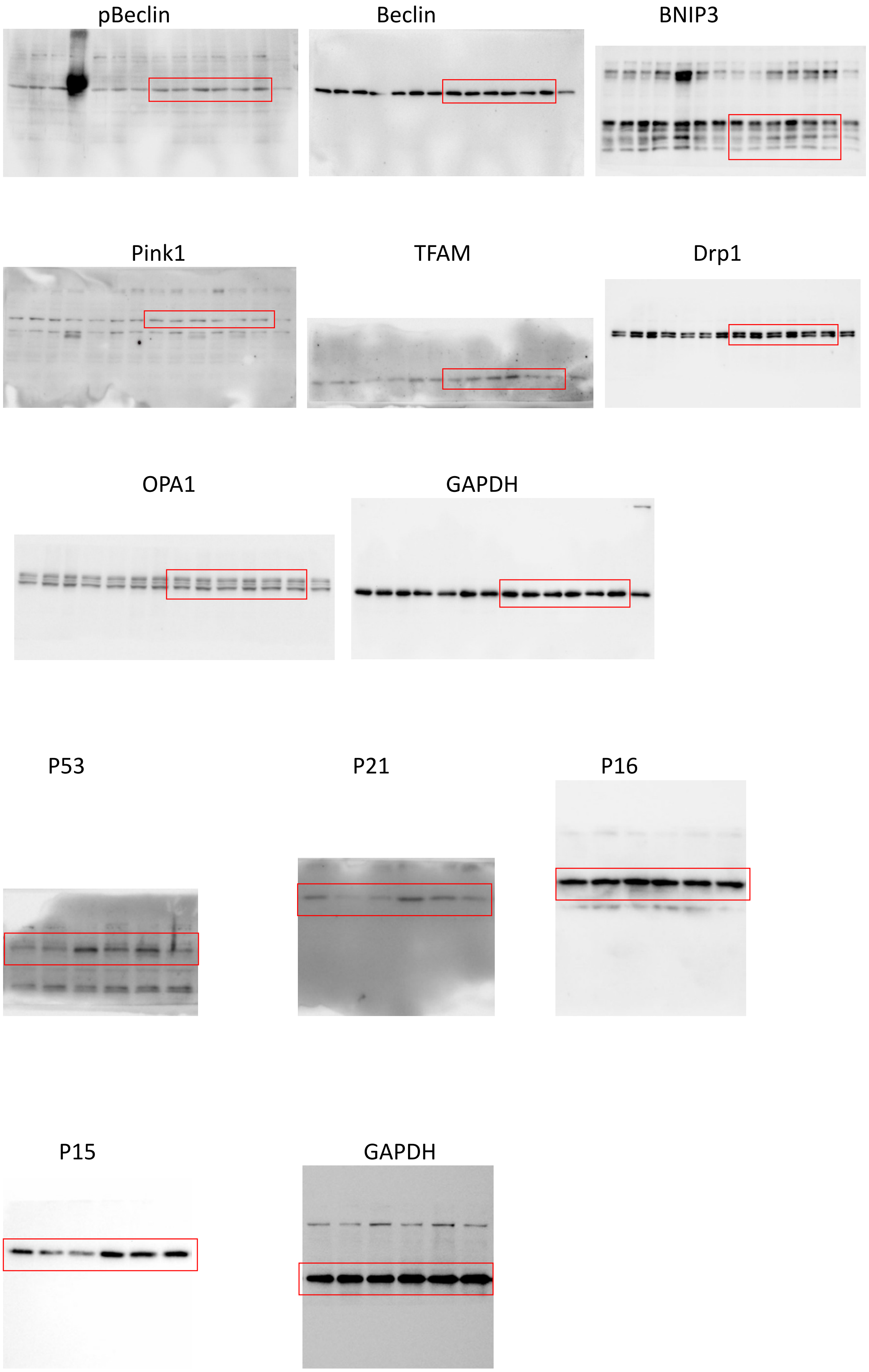
pBeclin
Beclin
BNIP3
Pink1
TFAM
Drp1
OPA1
GAPDH
P53
P21
P16
P15
GAPDH

Procaspase 3

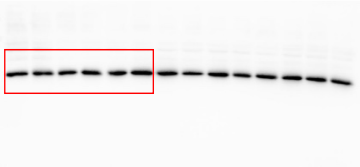

Caspase 3

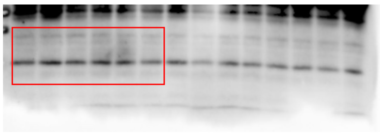

GAPDH

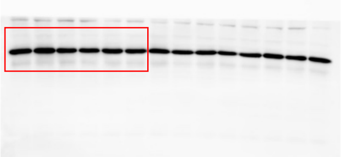

Puma

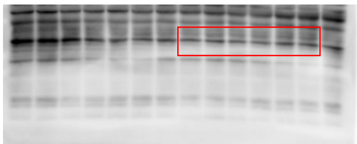

GAPDH

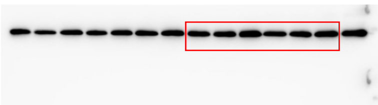

Lewis lung carcinoma model (LLC-MODEL)

pmTOR

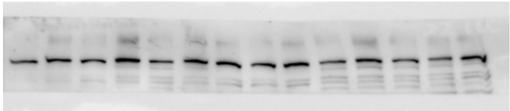

mTOR

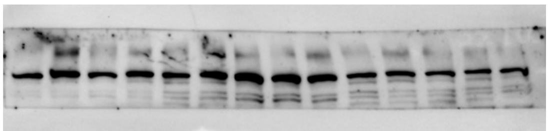

p4EBP1

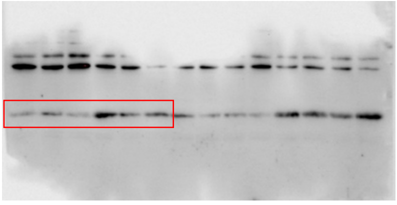

4EBP1

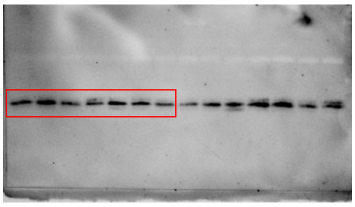

pS6

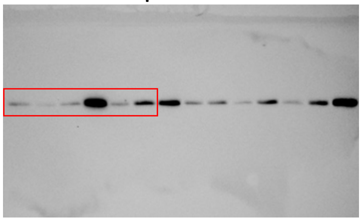

S6

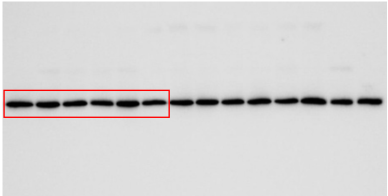

GAPDH

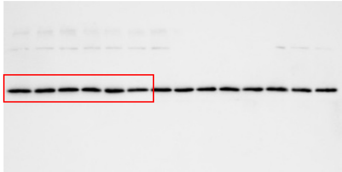

pGSK3

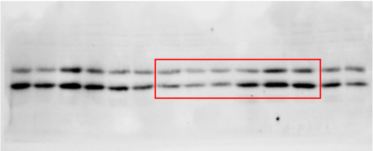

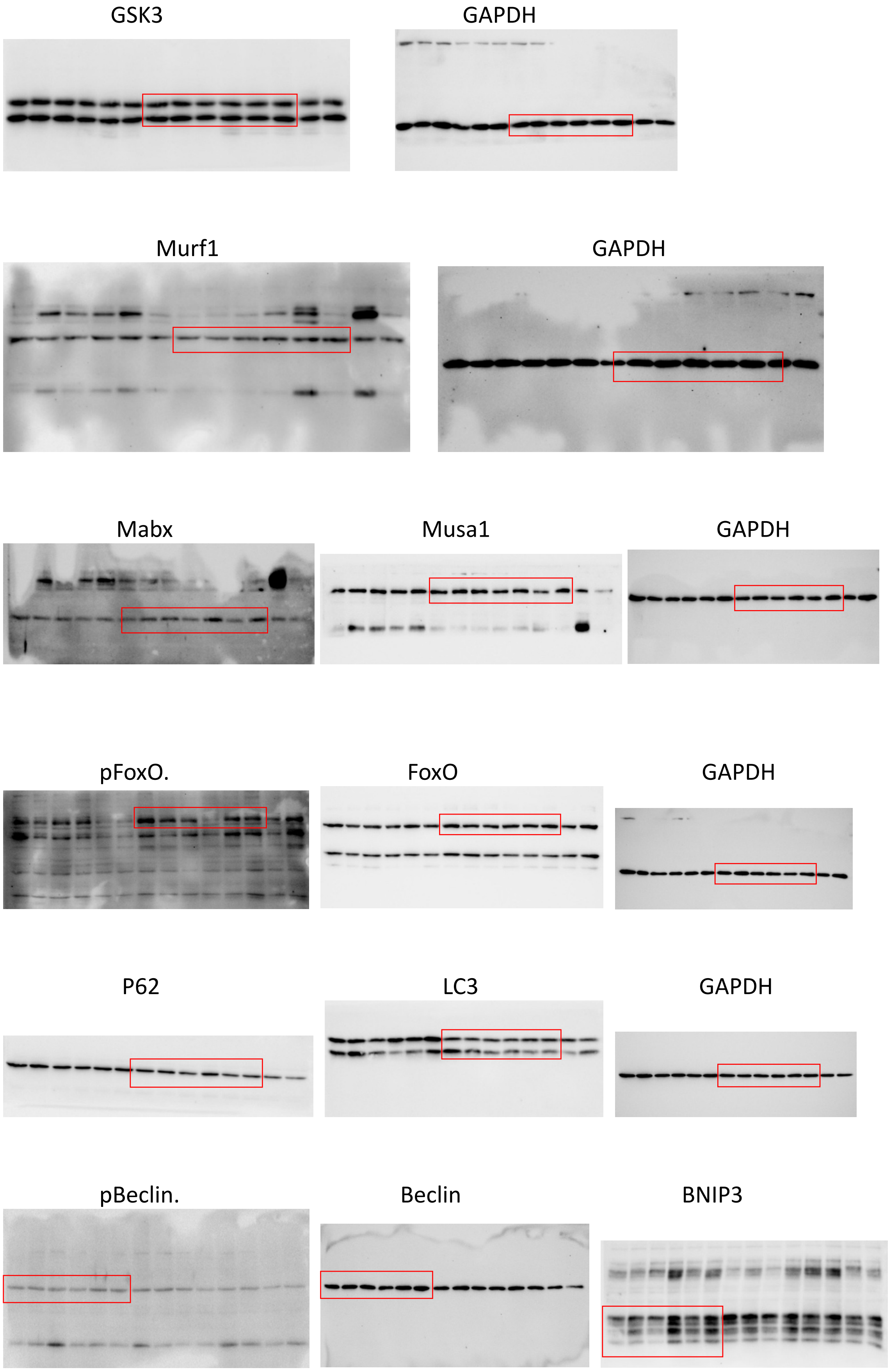

GSK3
GAPDH
Murf1
GAPDH
Mabx
Musa1
GAPDH
pFoxO.
FoxO
GAPDH
P62
LC3
GAPDH
pBeclin.
Beclin
BNIP3

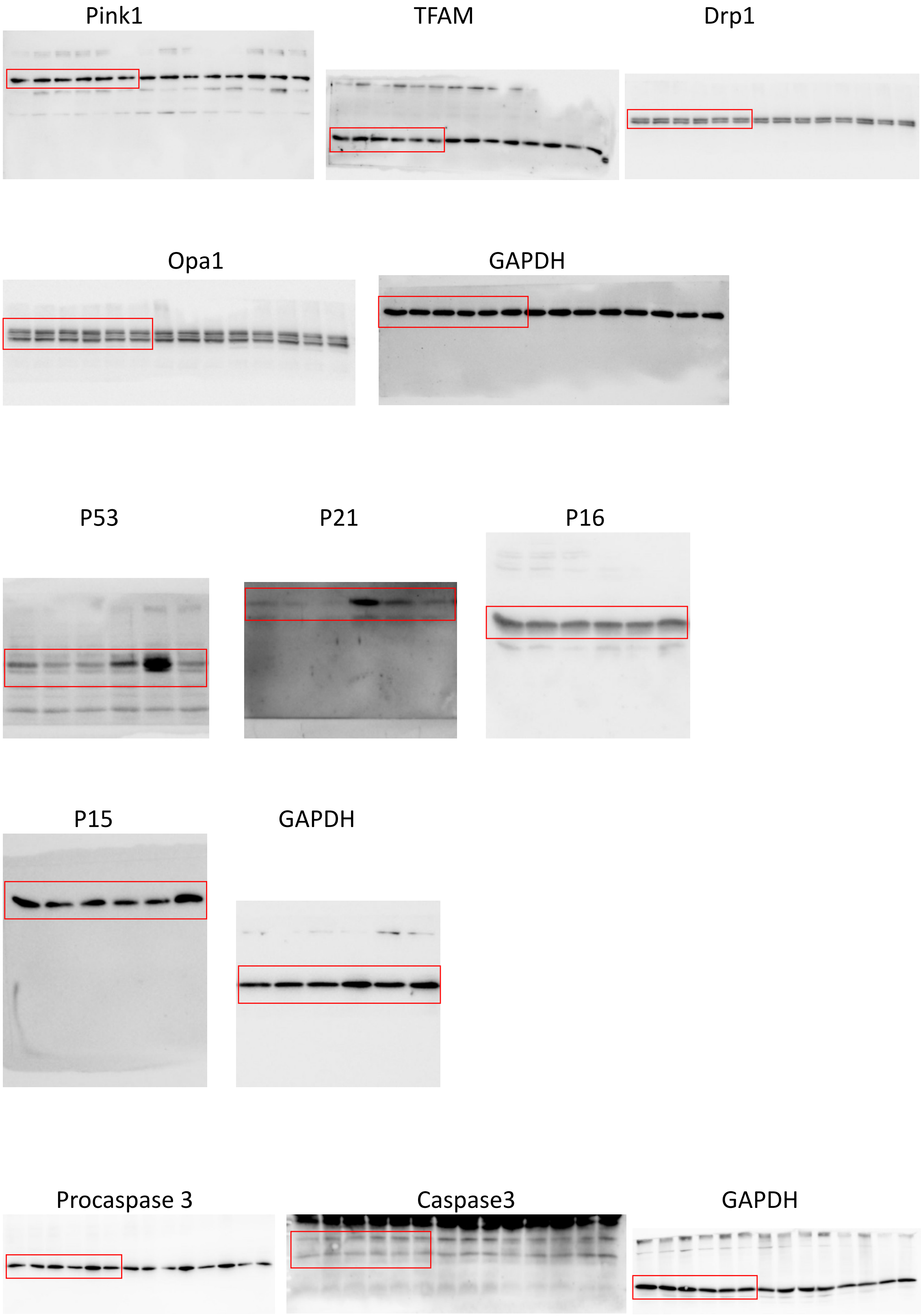
Pink1
TFAM
Drp1
Opa1
GAPDH
P53
P21
P16
P15
GAPDH
Procaspase 3
Caspase3
GAPDH

Puma

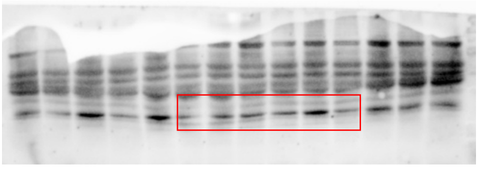

GAPDH

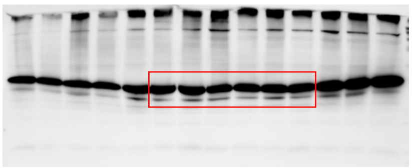

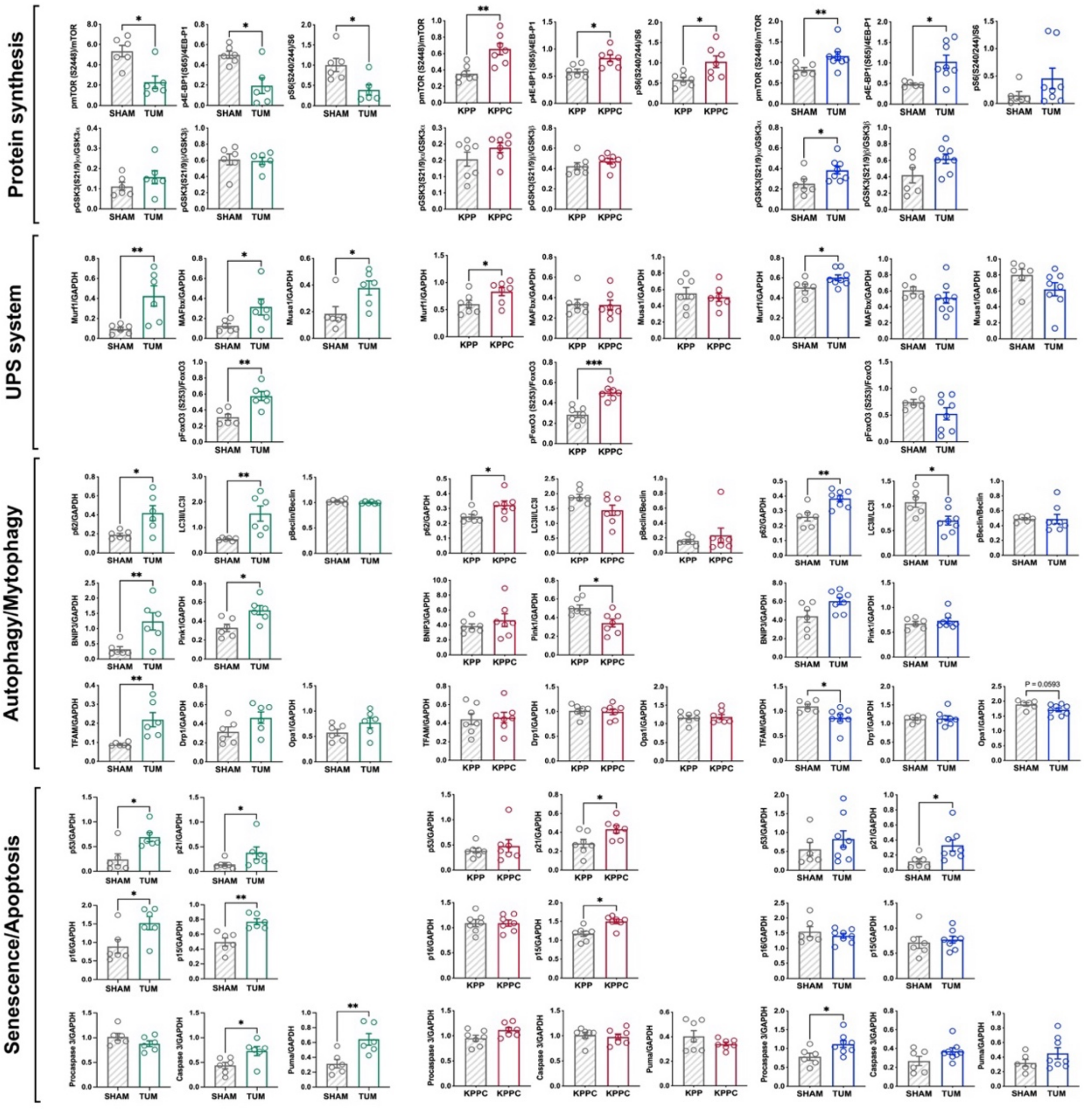
Protein synthesis
UPS system
Autophagy/Mytophagy
Senescence/Apoptosis
